# Evaluating bias in electronic health record data: using agent-based models to examine whether geographic disparities in community-acquired methicillin-resistant *Staphylococcus aureus* are due to differential health care–seeking behaviors

**DOI:** 10.1093/aje/kwae481

**Published:** 2025-01-06

**Authors:** Brittany L Morgan Bustamante, Jose Pablo Gomez-Vazquez, Carlos Gonzalez Crespo, Larissa May, Laura Fejerman, Beatriz Martínez-López

**Affiliations:** Environmental Health Sciences, School of Public Health, University of California, Berkeley, Berkeley, CA 4720, United States; Center for Animal Disease Modeling and Surveillance, Department of Veterinary Medicine and Epidemiology, School of Veterinary Medicine, University of California, Davis, Davis, CA 95616, United States; Center for Animal Disease Modeling and Surveillance, Department of Veterinary Medicine and Epidemiology, School of Veterinary Medicine, University of California, Davis, Davis, CA 95616, United States; Center for Animal Disease Modeling and Surveillance, Department of Veterinary Medicine and Epidemiology, School of Veterinary Medicine, University of California, Davis, Davis, CA 95616, United States; Emergency Medicine, School of Medicine, University of California, Davis, Davis, CA 95817, United States; Public Health Sciences, School of Medicine, University of California, Davis, Davis, CA 95616, United States; Center for Animal Disease Modeling and Surveillance, Department of Veterinary Medicine and Epidemiology, School of Veterinary Medicine, University of California, Davis, Davis, CA 95616, United States

**Keywords:** agent-based model, CA-MRSA, EHR disparities, bias analysis, geographic disparities

## Abstract

Electronic health records (EHRs) are increasingly used in public health research. However, biases may exist when using EHRs due to whether someone is captured in the data. Assessing the impact of bias in generating disparities identified with EHR data is difficult because information about health care–seeking behaviors is not included in the record. We developed an agent-based model (ABM) to simulate the health care–seeking behavior for community-acquired methicillin-resistant *Staphylococcus aureus* infection in a subregion of California. The ABM assumed no difference in prevalence across the study area. We modeled the health care–seeking process to see if geographic differences in prevalence would emerge from the ABM when only looking at those who sought treatment, matching empirical data. The ABM reproduced prevalence in observed data for 9 of the 21 geographies. Simulated differences in prevalence across geographies did not reach the magnitude in observed data, and spatial patterns had low to moderate agreement. Our results suggest that geographic disparities in the methicillin-resistant *Staphylococcus aureus* prevalence previously identified in California EHR data may be due to determinants beyond bias and health care–seeking behaviors. Future studies could adapt this model for other health outcomes by adjusting the health care–seeking behavior parameters and modifying the disease progression process.

**This article is part of a Special Collection on Cross-National Gerontology**.

## Introduction

Health disparities are preventable differences or gaps in health quality or outcomes that adversely affect socially and economically disadvantaged populations.^1^ Disparities exist among racial and ethnic minority groups, persons of low socioeconomic status, sexual and gender minorities, and geographic regions.^2^ While the drivers of disparities are complex and multifaceted, one potential mechanism for disparities in health observed across geography is differential access to health care and health care–seeking behaviors.^3^

Geographic differences in health care access and service utilization can be influenced by complex interactions between the distribution of social and environmental factors like income, insurance status, and the distance one must travel to obtain care.^4^^,^^5^ For instance, studies have shown that low-income communities have lower insurance coverage rates and are often primary health care shortage areas (HCSAs), restricting the available options for individuals seeking health care services.^4^ Even when individuals live near health care service providers and have insurance, low-income individuals may still delay seeking care or visit an emergency department (ED) rather than a primary care physician due to impaired primary care access or lower copayments in EDs.^6^ In the United States, EDs serve as a safety net, providing care for the uninsured and underinsured.^7^ In HCSAs and rural areas, EDs may be the only available health care service provider.^8^ The scarcity of primary care options and lower insurance rates may drive individuals in rural communities to delay seeking care or seek health care solely from nearby EDs.^9^ For research studies using electronic health record (EHR) data from EDs to describe health disparities, this differential access and utilization may result in the artifact of geographic disparities in observed health outcomes, with low-income and rural communities appearing to have a higher disease burden.

While EHRs are primarily used to manage clinical care, they are an increasingly rich data source in public health, and organizations are continuously working to improve access to records for research purposes. The California Office of Health Care Access and Information (HCAI) provides a yearly data set of patient discharges from every nonfederal ED licensed to provide care in California.^10^ The data set includes diagnostics in the form of International Classification of Diseases codes, demographic and payor information, treatments and procedures, and each patient’s zip code, which can be cross-walked to more meaningful neighborhood boundaries such as California’s Medical Service Study Areas (MSSAs).^11^ The data represent a comprehensive view of all ED visits in the state and can be used for epidemiologic explorations,^12^ informing policy initiatives,^13^ and examining patient characteristics or ED utilization trends.^14^ A recent epidemiologic analysis of these data showed measurable geographic disparities in community-acquired methicillin-resistant *Staphylococcus aureus* (CA-MRSA) infections across California MSSAs.^15^

When considering possible mechanisms contributing to unequal levels of infectious disease prevalence across geographies, differential health care access and differences in health care–seeking behaviors are particularly relevant for studies using EHR data.^2^^-^^5^ For example, a national survey of health care–seeking behaviors for skin and soft tissue infections found that 50% of individuals would not seek medical care if they developed a CA-MRSA infection.^16^ This suggests that approximately half of all cases could be missing in research studies using EHR data, and a higher disease burden would be identified in neighborhoods where patients sought treatment for their infection. If geographic differences in infection rates identified using EHR data merely reflect health care access and utilization behaviors, we would expect these spatial patterns to disappear if we could statistically control for them. Similarly, we would expect these patterns to emerge if we model access and seeking behaviors starting from a homogeneous geographic distribution of disease (ie, a hypothesized scenario where geographic disparities do not exist).

Unfortunately, EHR data capture information only on individuals who seek treatment, and we cannot directly test the influence of health care access and seeking behaviors. Agent-based modeling (ABM) is a promising method to explore the dynamic processes shaping the distribution of health outcomes, including how the spatial patterning of the built and social environments contributes to health disparities.^17^ ABM has been increasingly applied to population health problems due to its ability to study varying agents and scales; capture complex dynamic relationships, including feedback loops and adaptive systems; and model agency in decision-making.^18^ Previous research has used ABM to conduct hypothetical intervention studies, explore complex mechanisms and processes, and understand how the social and built environment shapes human behavior.^18^^,^^19^

We developed an ABM to assess whether health care–seeking behavior could impact the ecological association between geography and CA-MRSA infection rates.^20^^(p258)^ The ABM represents the counterfactual scenario with no geographic differences in infection and an entirely homogeneous disease distribution. Therefore, the model assumed no baseline geographic differences in CA-MRSA infection rates. We evaluated whether geographic disparities would emerge solely from individual health care–seeking behavior influenced by the social and built environment in which individuals reside. Despite the increasing use of EHR data in population health research and the acknowledgment of potential biases for studies using EHR data, to the best of our knowledge, an ABM exploring the influence of health care–seeking behaviors on observed disparities in the EHR has not been conducted.

## Methods

### ODD protocol summary

A complete, detailed model description, following the ODD (Overview, Design concepts, Details) protocol,^21^ is provided in the supplementary material (Appendix S1). The ABM simulates health care–seeking behaviors among CA-MRSA–infected individuals in a geographic subregion of Northern California to evaluate whether the association between geographic region and CA-MRSA prevalence observed in empirical data from California EDs can be explained by bias from differential health care–seeking patterns. Specifically, we addressed the following question: how does the geographic distribution of built and social determinants contribute to geographic inequalities in ED care-seeking behaviors and, consequently, observed disparities in CA-MRSA infections in ED EHRs? Our null hypothesis, which the ABM represents, was that there are no geographic disparities in CA-MRSA infection, and disparities observed in empirical ED data were due to health care–seeking behaviors. We tested our hypothesis by the ABM’s ability to reproduce geographic patterns in ED infection prevalence.

The ABM incorporated 2 entities: agents and MSSAs. The number of agents in each MSSA and their sociodemographic characteristics (insurance status/type and income above or below the federal poverty level [FPL]) were assigned using population proportions identified in census data, representing their probability distribution for the geographies included in the study. MSSAs are a geographic unit unique to California. They incorporate total population, socioeconomic, and demographic data provided by the US Census, combined with health care service and availability, to define area boundaries that maximize homogeneity in the social and structural environment. MSSAs are meant to represent where individuals within the area reasonably seek health care by accounting for commuting patterns and physical barriers like highways, mountains, and bodies of water.^22^ Using HCAI data, MSSAs, irregular polygonal regions on a map representing geographic areas, were initialized with a predetermined number of EDs, as well as primary care physicians, and designated as HCSA or not. A time step in the ABM represented 1 day, and simulations were run for 4 years to match the time span of the empirical data (2016 to 2019).

**Figure 1:**
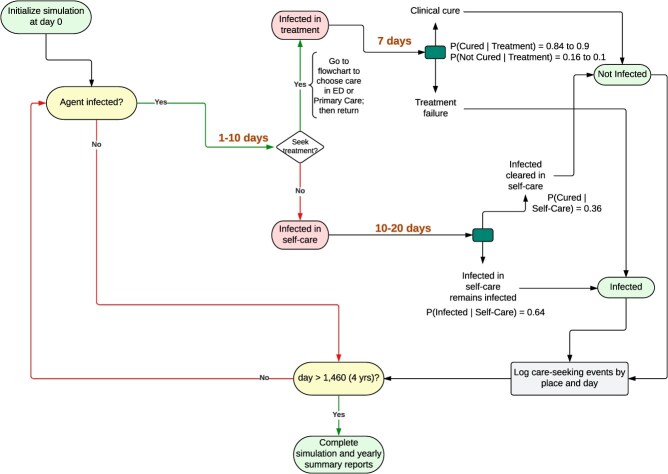
Agent-based model disease progression and transition submodel. Probabilities for these transitions were informed by the literature.

Unlike other infectious diseases commonly modeled, CA-MRSA has a colonized state. This means that individuals must carry the bacteria to be at risk of infection. At each time step, the ABM (1) created colonized agents characterized by unique sociodemographic variables (either uninsured and living below the FPL, public insurance and living below the FPL, uninsured and living above the FPL, public insurance and living above the FPL, or private insurance and living above the FPL) and (2) ran the “disease progression and transition” submodel (Figure 1). The disease progression and transition submodel is not conditional on sociodemographic variables and was informed by empirical literature to mirror the average progression of a CA-MRSA infection. In the disease progression and transition submodel, a proportion of colonized agents developed an infection, and the following actions occurred among the infected: (1) decide whether to seek treatment or self-care at home and, (2) if they sought treatment, whether to go to the ED or a primary care physician. Agents made these decisions based on their sociodemographic context and health care access. Agents seeking treatment may cross MSSA boundaries to receive care at an ED or primary care physician outside their neighborhood. However, cases were linked to the MSSA of residence rather than the MSSA in which they sought care, which matched the empirical data. The ABM was run for 5000 simulations, and we calibrated the model by varying the community colonization prevalence value (testing values from 0.6% to 7.4%) and a time decay parameter (testing values from 2% to 10%; equation (2)) until the percentage of agents seeking treatment in the ABM closely reflected the global probability of seeking treatment for CA-MRSA identified in the literature. We compared the median prevalence in ED cases across the 5000 simulation runs to the observed data.

At initialization, global parameter values for stochastic variables were chosen by drawing from categories identifying low, most likely, and high values (Table 1). The ABM assumed that agents not colonized with MRSA were not at risk of CA-MRSA infection, and at each time step, colonized agents were created according to a global, yearly prevalence value. If a colonized agent did not develop an infection after 6 months of being generated, the ABM assumed they became decolonized and removed them from the simulation.^23^ Agents could be infected more than once, which was modeled with a recurrence parameter. The values used for recurrence were based on a study reporting that 20% of previously infected patients with MRSA had a subsequent infection within 30 days and 36% over 120 days.^24^ To implement this in the ABM, we randomly initialized runs with a recurrence parameter reflecting the number of days the population was observed in the empirical study (ie, 0.20/30 [~0.007] or 0.36/120 [~0.003]).

**Table 1:** Global parameters for model initialization of agent disease state transition and health care–seeking behavior.

**Description**	**Value/range**	**Reference**
Proportion of population colonized with MRSA	1.5% (0.2%-7.4%)	^31^
Infection incidence	675/100 000 population	^16^ ^,^ ^32^
Time to decolonization for agents who do not develop an infection	6 months (median 1.8 months)	^23^
Period where an agent is infected but presymptomatic	4 (1-10) days	^33^
Global probability of seeking treatment for infection	50%	^16^
Probability of curing infection with treatment within 7 days	84.1%-90.5%	^34^
Probability of curing infection without treatment in 10-20 days^a^	36%	^32^
Probability of recurrent infection (over period at risk)	20-36%	^24^
Time period at risk of recurrent infection after clearing an infection with treatment^b^	64 (37-91) days	^35^
Time period at risk of recurrent infection after clearing an infection without treatment^b^	258 (191-325) days	^35^
*Assumptions for care-seeking behavior*		
Choosing to self-care if low-income (RR) (ref: ≥$60 000/year)	1.72 (1.07, 2.76)	^16^
Choosing to self-care if have a regular physician (RR) (ref: no regular physician)	0.64 (0.44, 0.95)	^16^
Choosing to self-care if had a previous infection (RR) (ref: no previous infection)	2.09 (1.40, 3.07)	^16^
Choosing to self-care if in health care shortage area (RR) (ref: living in area not designated health care shortage area)	1.22 (1.11, 1.33)	^8^
Percent decrease in the probability of choosing to self-care for each day an infection does not clear	2.5% ± 0.5%	
*Assumptions for choosing care in ED or primary care*		
Seeking treatment in ED with public insurance	42-55 visits per 100 individuals	^9^
Seeking treatment in ED with private insurance	16 visits per 100 individuals	^9^
Seeking treatment in ED with public insurance and living in poverty (OR) (ref: private insurance and above FPL)	1.5	^36^

Abbreviations: ED, emergency department; FPL, federal poverty level; MRSA, methicillin-resistant *Staphylococcus aureus*; OR, odds ratio; ref, reference group; RR, relative risk.

a Personal communication with infectious disease physician.

b Colonized but not active infection.

Deciding whether to seek treatment for their infection or self-care at home was modeled according to the Health Belief Model. The Health Belief Model is the theory that whether a person performs a health behavior is influenced by the degree to which they perceive the disease as threatening (perceived severity), the barriers and facilitators of an individual’s action (perceived barriers/facilitators), and the degree to which the behavior is believed to reduce the risk of an adverse health outcome (perceived benefit).^25^ The “health decision” equations (equations (1)-(3)) included these 3 original Health Belief Model constructs. Perceived barriers/facilitators included an agent’s income, insurance status, and care availability (ie, living in an MSSA with an ED or at least 1 primary care physician per 3000 residents), and perceived benefit was determined by an agent’s previous infection experience (if applicable) (equation (1)). Perceived severity was included in the model as a time decay function (*d*) (equation (2)). It was determined by infection duration (assuming the longer an infection went without clearing, the more complicated and severe it became, and the less likely someone was to continue self-care at home). The health decision process of choosing to self-care at home was controlled by the parameter ***sc*** (probability of self-care), which ranged from 0.0 to 1.0, multiplied by *d*. This value was used as the *p* parameter for a Bernoulli distribution, where 1 = self-care, 0 = seek treatment (equation (3)). If an agent sought treatment for their infection, they visited an ED or primary care physician according to the “seek treatment” submodel (Figure S2).


(1) \begin{equation*} sc=\frac{e^{0\,+\,0.54\left( Low\ income\right)-0.45(Insured)\,+\,0.74\left( Prior\& healed\right)\,+\,0.19(Unavailability)}}{1+{e}^{0\,+\,0.54\left( Low\ income\right)\,-0.45(Insured)\,+\,0.74\left( Prior\& healed\right)\,+\,0.19(Unavailability)}} \end{equation*}

Logged parameter values from Table 1:

- *Low income*: a binary indicator of whether an agent lives below the FPL (0 = no; 1 = yes)
- *Insured*: a binary indicator of whether an agent has insurance (0 = uninsured; 1 = insured)
- *Prior and healed*: a binary indicator of whether an agent had a previous infection that healed within 14 days, regardless of whether they sought treatment or self-cared (0 = no; 1 = yes)
- *Unavailability*: a binary indicator of whether an agent lives in an MSSA without an ED or enough primary care providers (ie, is a designated health care shortage area) (0 = no; 1 = yes)

- i) The resulting value was then multiplied by a time decay function^17^ representing perceived severity:

(2) \begin{equation*} d={e}^{-\beta t} \end{equation*}

- where $t$ is the time in days the agent has been infected, and $\beta$ is the time decay parameter (0.025 ~ 2.5% reduction per day).

- ii) The $sc\ast d$ value was then sampled from a Bernoulli distribution:

(3) \begin{equation*} x=\sim Bernoulli(p) \end{equation*}

- where $p=(sc)\ast (d)$ and *x* = 1 for self-care; *x* = 0 for seek treatment.

### Analysis of simulated data

We compared the output of the ABM to ED data from HCAI between 2016 and 2019. We used Markov chain Monte Carlo pseudo-*P* values to identify MSSAs, where the number of ED visits for infection from the ABM (expected ED) was significantly less than the observed data (observed ED). Random forest algorithms were used to explore and rank the most influential parameters from the ABM across 1000 simulation runs, and classification and regression trees were used to obtain a graphical representation of the outcomes, following the global sensitivity analysis process described by Harper et al.^26^ The geographic distribution of the expected 4-year risk of CA-MRSA infection presenting in the ED, calculated as the number of infected agents seeking treatment in the ED per 100 000 MSSA population over the 4 years from the ABM, was visually compared to the geographic distribution from the observed ED data. MSSAs were also ranked from highest to lowest prevalence based on the ABM results and the observed data. The rankings were compared using Kendall’s coefficient of concordance (*W*) to measure agreement in the patterning of infection prevalence between the ABM and observed data, irrespective of the difference in the magnitude of infection prevalence. Partial least squares (PLS) was used to conduct a relative importance analysis and identify the key drivers in explaining MSSA-level observed ED prevalence.^27^

Variables in the PLS included the percentage of the population identifying as non-Hispanic White, meeting the federal definition for living in a crowded household, living below 100% of the FPL, under 5 years of age, between 18 and 64 years of age, identifying as an immigrant, the average level of environmental degradation in the MSSA, and the expected prevalence output from the ABM for each MSSA (representing the proportion of the observed ED prevalence potentially explained by health care–seeking behavior). The demographic variables were taken from the 2019 American Community Survey, and the average level of environmental degradation was obtained from the California Office of Environmental Health Hazard’s CalEnviroScreen 4.0.^28^ All variables were bounded between 1:100 and scaled before analysis. Code for reproducing the ABM results is available at https://github.com/cadms/mrsa-abm. The ABM was implemented in the GAMA Platform, a simulation platform and open-source modeling environment for creating spatially explicit agent-based simulations,^29^ and the model outcomes were analyzed in R version 4.2.1.^30^

## Results

In the HCAI empirical data, 10 301 patients were diagnosed with CA-MRSA in the study region between 2016 and 2019, with prevalence values ranging from 1.4% to 11.5%. Most residents in the study area had either public or private health insurance, and a little over half of the MSSAs were HCSAs (57.1%). A detailed overview of the MSSA characteristics and the residents residing in each MSSA included in the study can be found in the supplemental material (Table S2).

In the ABM, the number of CA-MRSA cases presenting in the ED was influenced by community MRSA colonization prevalence. An assumed community colonization prevalence of 3% produced a 4-year risk of infection presenting in the ED similar to the observed data (0.108 cases per 100 000), while a community prevalence of 7.4% overestimated the observed 4-year risk of infection presenting in the ED (0.134 cases per 100 000). We compared the results from runs with the 3% community colonization value, a colonization length of 200 days, and a recurrence parameter of 0.007 to the observed data.

The ABM produced only slight heterogeneity in MRSA prevalence across the MSSAs, ranging from >0.08% to 0.15%, appropriately demonstrating the null hypothesis of a scenario with no true geographic disparities (Figure S3a). About 60% (56.33% to 61.95%) of infected agents chose to seek treatment at some time during their infection (Figure S3b). A higher percentage of agents were treated in the ED than by a primary care physician (Figure S3d). Spatial patterning appeared in infection prevalence among agents seeking treatment in the ED (Figure S3c). However, prevalence did not vary greatly by MSSA (3.71% to 8.34%).

**Figure 2:**
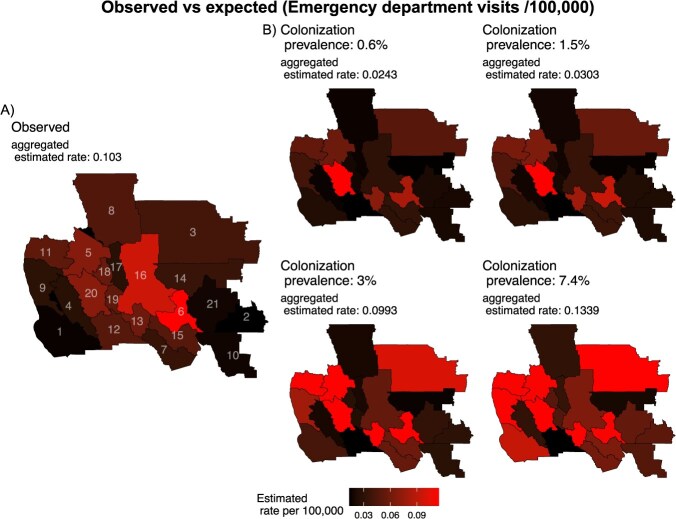
Observed 4-year risk of community-acquired methicillin-resistant *Staphylococcus aureus* (MRSA) infections (per 100 000) in empirical emergency department data from California (A) compared to agent-based model–produced infection risk presenting in the emergency department at different community MRSA colonization prevalence values (B). ^*^Numbers on the observed emergency department map are Medical Service Study Area IDs.

In the observed ED data, the highest 4-year risk of infection was seen in central MSSAs (Figure 2A), and the overall aggregated 4-year risk of infection presenting in the ED was 0.103 cases per 100 000. The ABM consistently produced high infection risk in EDs in eastern MSSAs and 1 northwestern MSSA (Figure 2B). Several central MSSAs had significantly lower expected infection risk in EDs than the observed ED data (Figure S4).

Table 2 presents the MSSAs with a rank value from highest to lowest infection prevalence from the ABM and observed ED data. The ABM estimated the infection prevalence identified in the observed data within 2% for 42.9% of the MSSAs. When comparing prevalence relative to other MSSAs, irrespective of the difference in magnitude, the ABM ranked 9 of the 21 MSSAs (43%) within an absolute value of 5 positions of what was observed in the empirical data and 4 of the 21 MSSAs (19%) within an absolute value of 1 position. Kendall’s *W* indicated low to moderate agreement between the MSSA ranking order in the observed ED and the ABM expected ED data (*W* = 0.51, *P* = .43).

**Table 2:** Community-acquired methicillin-resistant *Staphylococcus aureus* prevalence and geography ranking for California communities from highest to lowest prevalence values for observed emergency department data and the agent-based model output.^a^

**MSSA**	**ID**	**Population**	**Observed cases**	**ABM cases**	**Observed case prevalence (%)**	**ABM case prevalence (%)**	**Observed rank**	**ABM rank**	**Rank difference**
Anchor Bay/Gualala/Manchester/Point Arena	1	4188	67	347	1.60	8.29	20	4	16
Arbuckle/Grimes	2	5647	79	242	1.40	4.29	21	18	3
Artois Elk Creek/Glenn/Grindstone Indian Rancheria/Willows	3	10 668	441	888	4.13	8.32	12	3	9
Boonville/Navarro/Philo/ Yorkville	4	3023	85	151	2.81	5.00	17	12	5
Brooktrails/Pine Mountain/Willits	5	12 690	866	1013	6.82	7.98	4	6	2
Clearlake/Clearlake Oaks	6	19 553	2244	1010.5	11.48	5.17	1	11	10
Cobb/Hidden Valley/Middleton	7	9957	350	490	3.52	4.92	14	13	1
Covelo/Dos Rios	8	2674	127	150	4.75	5.61	11	8	3
Elk/Little River/Mendocino	9	8345	250	696	3.00	8.34	15	1	14
Esparto/Rumsey	10	5722	103	234.5	1.80	4.10	19	20	1
Fort Bragg/Westport	11	12 078	636	1006	5.27	8.33	8	2	6
Hopland	12	2051	100	88	4.88	4.29	10	17	7
Kelseyville/Lakeport	13	16 029	942	1013	5.88	6.32	5	7	2
Lower Lake	15	9787	478	512	4.88	5.23	9	10	1
Lucerne/Nice/Upper Lake	16	8869	852	473	9.61	5.33	2	9	7
Maxwell/Princeton/Stonyford	14	2252	81	110	3.60	4.88	13	14	1
Potter Valley	17	1869	53	154	2.84	8.24	16	5	11
Redwood Valley	18	5322	300	224	5.64	4.21	6	19	13
Talmage	19	4097	218	197	5.32	4.81	7	15	8
Ukiah	20	27 345	1918	1014	7.01	3.71	3	21	18
Williams	21	5963	111	271	1.86	4.54	18	16	2

Abbreviations: ABM, agent-based model; MSSA, Medical Service Study Area.

a Population = population total. Rank difference = absolute value difference between ranking in ABM and ranking in observed data.

PLS analysis identified 3 independent variables as positive predictors that explained the observed ED prevalence. The most important variables were the percentage of residents living below 100% of the FPL, the percentage of residents identifying as non-Hispanic White, and the average environmental degradation score (Figure S5a, b). Combined, these variables explained 54.7% of the observed CA-MRSA ED prevalence variability across the MSSAs.

### Sensitivity analysis

Random forest algorithms identified the length of time colonized and community colonization prevalence as the most influential parameters on the outcome, ED visits. The percentage of agents seeking treatment for infection was most influenced by how long agents could be colonized without an infection before being removed from the simulation and the probability of having a recurrent infection. In scenarios with high colonization length and infection recurrence, the treatment probability was 58%. In contrast, in scenarios where the colonization length and infection recurrence were low, the probability of seeking treatment was 89%. The influence of the parameters and the interactions between them are presented in Figure S6.

## Discussion

Studies using EHR data are susceptible to biases that may distort observed associations between disparity populations and disease burden estimates due to a lack of representativeness of the study sample to the population of interest. EHR data only record encounters initiated by the patient choosing to visit a health care facility, and understanding the mechanisms driving whether a patient is captured in the EHR is difficult. This study simulated health care–seeking behaviors among CA-MRSA–infected agents to evaluate whether bias arising from differential health care–seeking patterns could explain small area-level inequalities observed in empirical data from California EDs. The emergent property of the ABM (spatial patterning of infections presenting in the ED) did not replicate the observed ED data. Kendall’s *W* was insignificant, meaning we failed to reject the null hypothesis that no agreement exists between the prevalence ranking/distribution in the ABM and observed ED data. Our results suggest that health care–seeking behavior is unlikely solely responsible for the geographic disparities identified in the observed data, and additional factors likely contribute to the geographic distribution of CA-MRSA infections.

We conducted a PLS analysis to evaluate what factors could contribute to the geographic disparities in infection prevalence. The percent poverty, percent non-Hispanic White, and average environmental degradation score were identified as variables explaining variance in observed CA-MRSA ED prevalence. A strong correlation exists between environmental degradation and socioeconomic disadvantage. Environmental determinants, such as water quality, pollution, and local temperature, have previously explained variability in infectious disease patterns.^37^^-^^39^ A recent analysis of these same data showed that area-level poverty was associated with infection incidence in individuals across California, and this relationship was partially mediated by average environmental degradation in the MSSA.^40^ While the current analysis was on a subsample of the larger California data set and conducted at an ecological level, the consistency in identifying an association between poverty, environmental degradation, and CA-MRSA infection across studies provides further evidence of the important role the natural environment may play in CA-MRSA infections. Future work should expand our PLS by evaluating the role of biologically plausible sources of environmental contamination in CA-MRSA transmission dynamics rather than looking at average environmental degradation, helping to inform mitigation or educational campaigns that may reduce community-level exposure.

Our ABM, guided by the Health Belief Model, incorporated factors hypothesized to contribute most significantly to health care–seeking behaviors. Our spatial unit of analysis, MSSA, is designed to maximize homogeneity in the sociostructural environment and best represent where individuals in each area reasonably seek health care. However, it is impossible to model or account for all factors influencing an individual’s propensity to seek health care in a single ABM. Several factors, such as age, sex, and physical access, were considered when developing MSSA boundaries, but other determinants, such as stigma, health care discrimination, or language barriers, were not.^41^ We tried to assess the importance of this omission in the PLS and found that the percentage of the population identifying as an immigrant was not a variable explaining variance in observed CA-MRSA ED prevalence. However, it is possible these important drivers influence the likelihood that someone seeks treatment for CA-MRSA and that our ABM is not fully capturing the complexity of the decision-making process for MSSAs with a high percentage of immigrant, non-English-speaking, or racial and ethnic minority populations. Further, there are other ways to assess the catchment of a health care system that could be considered and incorporated in future ABMs, other than using MSSA, including travel time or distance-based measures,^42^ patient population proportions,^43^^,^^44^ and k-means clustering.^45^

The ABM was informed by the literature and empirical evidence where possible. However, the most influential parameters were the time an agent was colonized and community colonization prevalence, which are the parameters with the most uncertainty. Individuals can be asymptomatically colonized with MRSA, and few studies conduct random, representative sampling for MRSA colonization.^31^ The most likely prevalence value identified in the literature (1.5%) was from a 2003-2004 National Health and Nutrition Examination Survey random sample, which may be outdated.^46^ We tested prevalence values as low as 0.6% and as high as 7.45% and found that values around 3% most closely replicated the overall 4-year risk of infection presenting in EDs in the observed data. Other studies have estimated that around 50% of individuals infected with MRSA will seek treatment for their infection. In our ABM, the percentage of agents seeking treatment most closely mirrored this estimate in scenarios with high colonization length and infection recurrence.

Further, due to computational restraints, we ran the simulations only for a subpopulation of California. Other areas of the state with different demographics may produce different results. Finally, there are other sources of potential bias in EHR data, including studies using International Classification of Diseases codes to document disease status.^47^ The goal of this article was only to evaluate the influence of bias due to health care–seeking behaviors, as this type of bias is more difficult to evaluate and cannot be easily controlled or mitigated statistically.

A key strength of ABM is the ability to show how complex outcomes emerge from a simple set of rules. Previous studies have suggested that bias due to health care–seeking behaviors may explain disparities observed in EHR data.^48^^,^^49^ While we had to make simplifications and assumptions to feasibly model health care–seeking behavior, a key advancement in this article is the development of an ABM that can test this hypothesis in a controlled environment. Our study started with a homogeneous disease distribution, demonstrating little to no geographic disparities in CA-MRSA infection, and the spatial patterning of CA-MRSA ED prevalence had low to moderate agreement with the observed data. While we cannot entirely rule out the possibility that our ABM is misspecified and that health care–seeking behaviors significantly contribute to the observed geographic disparities, the current study leads us to conclude that factors beyond health care–seeking behaviors may be responsible for geographic disparities identified in the observed ED data. Future studies could adapt this model for other health outcomes by adjusting the health care–seeking behavior parameters and modifying the disease progression process.

## Supplementary Material

Web_Material_kwae481

## Data Availability

The code for reproducing the ABM results and analyzing the output is available at https://github.com/cadms/mrsa-abm.
