# Supplementary material for: Evaluating bias in electronic health record data: using agent-based models to examine whether geographic disparities in community-acquired methicillin-resistant Staphylococcus aureus are due to differential health care–seeking behaviors
Source: Am J Epidemiol. 2025 Jan 6;194(12):3407–15. doi: 10.1093/aje/kwae481 (PMC12671960; doi:10.1093/aje/kwae481)
Supplement: Web_Material_kwae481 [file web_material_kwae481.zip › Supplemental_RR3.docx]

**Title:** Evaluating bias in electronic health record data: using agent-based models to examine whether geographic disparities in community-acquired Methicillin-resistant *Staphylococcus aureus* are due to differential healthcare-seeking behaviors

**Authors**: Brittany L. Morgan Bustamante^1,2^, Jose Pablo Gomez-Vazquez^2^, Carlos Gonzalez Crespo^2^, Larissa May^3^, Laura Fejerman^4^, Beatriz Martínez-López^2^

**Affiliations**: ^1^Environmental Health Sciences, School of Public Health, University of California, Berkeley, Berkeley, CA, United States; ^2^Center for Animal Disease Modeling and Surveillance, Department of Veterinary Medicine and Epidemiology, School of Veterinary Medicine, University of California, Davis, Davis, CA, United States; 3Emergency Medicine, School of Medicine, University of California, Davis, Davis, CA, United States; ^4^Public Health Sciences, School of Medicine, University of California, Davis, Davis, CA, United States

**Table of Contents**

**Appendix S1**: ODD Protocol..........................................................................................................2

**Table S1**: Global parameters for model initialization of agent disease state transition and healthcare seeking behavior.............................................................................................................8

**Figure S1**: Agent-based model disease progression and transition submodel. Probabilities for these transitions were informed by the literature...........................................................................10

**Figure S2**: Seek Treatment Submodel..........................................................................................13

**Figures S3a-3d**: Agent-based model output for CA-MRSA infection outcomes.........................14

**Figure S4**: MSSAs with statistically significantly fewer CA-MRSA cases produced from the ABM than observed in the empirical ED data as identified using Markov Chain Monte Carlo pseudo-*P-*values.............................................................................................................................15

**Figures S5a-b**: Results from PLS relative importance analysis...................................................16

**Figure S6**: Global sensitivity analysis (GSA) for CA-MRSA infection prevalence in emergency department......................................................................................................................................17

**Table S2**: Characteristics of MSSAs and MSSA residents in the study area between 2016-2019................................................................................................................................................18

**Appendix S1: ODD Protocol**

### **Purpose**

### The overall purpose of our model is to simulate healthcare-seeking behaviors among CA-MRSA-infected individuals to evaluate whether small area-level inequalities observed in empirical data from California EDs can be explained by bias due to differential healthcare-seeking patterns. Specifically, we are addressing the following question: how does the geographic distribution of built and social determinants contribute to geographic inequalities in ED care-seeking behaviors and, consequently, observed disparities in CA-MRSA infections in ED EHR? Our null hypothesis, which the ABM represents, is that there are no geographic disparities in CA-MRSA infection, and disparities observed in empirical ED data are due to biases stemming from healthcare-seeking behaviors. We evaluate our model by its ability to reproduce geographic patterns in ED infection prevalence.

### **Entities, state variables, and scales**

### **Agents/Individuals:** Agents in the model include individuals and MSSAs. *Individuals* are the active entities of the model and represent the susceptible or at-risk population in each MSSA. The model assumes that individuals not colonized with MRSA are not at risk of a CA-MRSA infection and that decisions regarding whether to seek treatment for their infection within a healthcare facility are determined by their state variables. The state variables of an agent are their income status (above or below FPL) and insurance status/type (uninsured, public insurance, private insurance). MSSAs are irregular polygon patches of a choropleth map. The state variables of the MSSA are the coordinates of its location, the distribution of PC providers and EDs within its boundary, and a list of the agents occupying it. The model assumes a homogenous CA-MRSA colonization and infection rate across the MSSAs and that individuals are less likely to seek treatment if there is no healthcare facility in their MSSA.

### **Scales:** Each time step in the model represents one day, and simulations will run for 4 years (1,460 days).

### **Process overview and scheduling**

### In each time step, colonized and infected individuals in each MSSA are randomly generated according to global parameter values. All agents begin the simulation in the colonized state and move through the “disease progression and transition” submodel (described below). When an individual develops an infection, they enter the “health decision” submodel (described below) and decide whether to seek treatment or self-care at home according to an objective parameter (*sc*, described below). The parameter *sc* represents an infected individual's probability of self-caring at home and is estimated per infected agent, per time step of the simulation based on agent state values**.** If they seek treatment, they decide to visit the ED or a PC by the “seek treatment” submodel (described below). Those who seek treatment have their infection cured at a higher probability and quicker than individuals who self-care. If they self-care at home, they have a higher probability of remaining infected and clearing the infection takes longer.

### **Design concepts**

### **Basic principles:** The basic principle of this model is the idea it is designed to illustrate: the extent to which geographic disparities in infection rates can arise from healthcare-seeking behaviors and decisions. Do geographic disparities in infection rates in observed data merely reflect a self-selection process among those seeking treatment for their infections? Understanding this principle can be critical for the exploration of geographic disparities determinants and the use of EHR data in infectious disease epidemiology.

### **Emergence:** The results we are interested in are the number of CA-MRSA infections seeking treatment in the ED. If we assume the disparities observed in empirical data are due to individual-level decision-making regarding when and where to seek treatment, then the emergent property of this model will recreate the geographic distributions of infection observed in empirical data. Alternatively, if geographic disparities observed in ED EHR data are due to drivers beyond whether someone chooses to seek care in an ED, the emergent property of this model will not recreate the geographic distributions of infections in the empirical data.

### **Adaptation:** Infected individuals have one adaptive behavior: deciding whether to seek treatment for their infection or not and, if so, at a PC office or in the ED. The adaptive behavior of whether to seek treatment is modeled as direct objective seeking: an individual seeks treatment for their infection if their objective measure (Objectives, below) is below a threshold. The threshold is a model parameter named *sc*. This submodel (the “health decision” submodel) is based on the Health Belief Model (HBM). The HBM proposes that whether a person performs a health behavior is influenced by the degree to which they perceive the disease as threatening (perceived severity) and the degree to which the health behavior is believed to effectively reduce the risk of a negative health outcome (perceived benefit).^1^ Perceived severity is determined by whether someone believes they are susceptible to the disease and how severe they believe it is or will be if they develop the disease. Perceived benefit is not only focused on whether the person believes that a behavior is useful but what the behavior will cost the individual in terms of money, time, and effort (perceived barriers). These basic principles were included in the “health decision” submodel. If an individual does decide to seek treatment, they choose whether to visit an ED or PC using a flow diagram described below (the “seek treatment” submodel).

### **Objectives:** The infected agents’ objective measure is the parameter *sc*, representing the probability of self-caring a home. *Sc* is a function of the severity of the individuals’ infection, their previous experience with infections, and the resources readily available to them (economic - income/insurance; availability - income/travel distance)

### **Learning:** The adaptive behavior of individuals – deciding whether to seek treatment for their infection or self-care at home – is modeled using an approach that includes learning. This submodel (“Health Decision submodel” explained below) assumes individuals learn from their previous experience with infections. If they had a previous infection that cleared quickly, with or without treatment, they are less likely to seek treatment for their current infection.^2^ Alternatively, if they sought treatment and had treatment failure or self-cared at home and developed a severe infection, they might more readily seek treatment for their current infection.

### **Sensing:** Individuals are assumed to know the healthcare facilities available, if any, in their MSSA.

### **Stochasticity:** Stochasticity is used in initializing the model (*Initialization*, below). First, the model is initialized stochastically in such a way that (a) the colonization prevalence, (b) the pre-symptomatic period, (c) the probability of clearing an infection, (d) the probability of recurrent infection, (e) the time at risk for recurrent infection, (f) the threshold value for the *sc* parameter, and (g) the probability of choosing to seek treatment at an ED or PC for each state characteristic are all stochastically chosen from a range of possible values. These initialization methods are stochastic, so each model run produces different results in line with what was identified in the literature.

### **Observation:** The observational data collected from the model for analysis is the MSSA population-level prevalence of CA-MRSA and the MSSA population-level prevalence of CA-MRSA infections treated in EDs. The graphical output of the model includes incidence choropleth maps. Pseudo-*P*-values are calculated to compare the expected values (from the ABM) to the observed values in the empirical data. Random forests are used to rank the most influential parameters.

### **Initialization**

### Individual agent initialization: At initialization, global parameter values for stochastic variables are chosen by drawing from either probability distributions describing their variability or categories identifying low, most likely, and high values (**Table S1**). Unlike other infectious diseases commonly modeled, CA-MRSA has a colonized state. Meaning individuals must carry the bacteria to be at risk of infection. At the beginning of the simulation, individuals colonized with MRSA are randomly generated based on the global colonization prevalence value chosen. Each individual’s state variables (income above or below FPL and insurance status/type) are initialized using MSSA-level population proportions identified in census data, representing their probability distribution.

### MSSA agent initialization: MSSAs are initialized with a pre-determined number of EDs and PC physicians based on empirical data. MSSA positions and shapes are initialized using spatial data and shapefiles.

**Table S1: Global parameters for model initialization of agent disease state transition and healthcare-seeking behavior**

| **Description** | **Value/Range** | **Reference** |
| --- | --- | --- |
| Proportion of population colonized with MRSA | 1.5% (0.2-7.4%) | 3 |
| Infection incidence | 675/100,000 population | 2,4 |
| Time to decolonization for agents who do not develop an infection | 6 months  (median 1.8 months) | 5 |
| Period where an agent is infected, but pre-symptomatic | 4 (1-10) days | 6 |
| Global probability of seeking treatment for infection | 50% | 2 |
| Probability of curing infection with treatment within 7 days | 84.1%-90.5% | 7 |
| Probability of curing infection without treatment in 10-20 days^+^ | 36% | 4 |
| Probability of recurrent infection (over period at risk) | 20-36% | 8 |
| Time period at risk of recurrent infection after clearing an infection with treatment^*^ | 64 (37-91) days | 9 |
| Time period at risk of recurrent infection after clearing an infection without treatment^*^ | 258 (191-325) days | 9 |
| *Assumptions for Care Seeking Behavior* | | |
| Choosing to self-care if low-income (RR) (ref: ≥ $60,000/year) | 1.72 (1.07, 2.76) | 2 |
| Choosing to self-care if have a regular physician (RR) (ref: no regular physician) | 0.64 (0.44, 0.95) | 2 |
| Choosing to self-care if had a previous infection (RR) (ref: no previous infection) | 2.09 (1.40, 3.07) | 2 |
| Choosing to self-care if in Healthcare Shortage Area (RR) (ref: living in area not designated healthcare shortage area) | 1.22 (1.11, 1.33) | 10 |
| Percent decrease in the probability of choosing to self-care for each day an infection does not clear | 2.5% ± 0.5% |  |
| *Assumptions for Choosing Care in ED or Primary Care* | | |
| Seeking treatment in ED with public insurance | 42-55 visits per 100 individuals | 11 |
| Seeking treatment in ED with private insurance | 16 visits per 100 individuals | 11 |
| Seeking treatment in ED with public insurance & living in poverty (OR) (ref: private insurance & above FPL) | 1.5 | 12 |

RR = Relative Risk; OR = Odds Ratio; ref = reference group; FPL = federal poverty level; ^+^personal communication with infectious disease physician; ^*^colonized but not active infection

1. **Input data**

One input data file is used in the model. The file contains data collated from various sources: the proportion of individual agents' state characteristics for each MSSA identified from US Census data and California Health Interview Survey, the location and shape of MSSAs, and the number of EDs and PC physicians in each MSSA derived from the California Department of Health Care Access and Information.

### **Submodels**

### The following submodels are run to simulate infected individuals' disease progression and healthcare-seeking behavior process. Some submodels are run every time step while others are run only when certain conditions are met. For example, the “seek treatment” submodel is run once an infected individual decides to seek treatment for their infection.

### **Disease progression and transition**: At each time step, individuals colonized with MRSA are randomly generated in each MSSA based on the homogeneous application of the global colonization prevalence value chosen for that simulation run. With each subsequent time step, colonized individuals have a probability of infection based on a global incidence value identified in the literature and corrected for underreporting (675/100,000 individuals).^2,4^ When individuals clear their infection, they remain at increased risk for re-infection for 64 (37-91) days if they received medical treatment and 258 (191-325) days if they did not.^9^ To conserve computational power, if an agent has been colonized for six months without developing an infection, they are removed from the simulation and no longer followed. This aligns with evidence demonstrating that most colonized individuals are decolonized within six months barring medical treatment.^5^ As visualized below, the disease progression and transition submodel defines how an infected individual’s disease progresses through time, depending on whether they seek treatment or self-care at home.

**Figure S1**: **Agent-based model disease progression and transition submodel. Probabilities for these transitions were informed by the literature.**


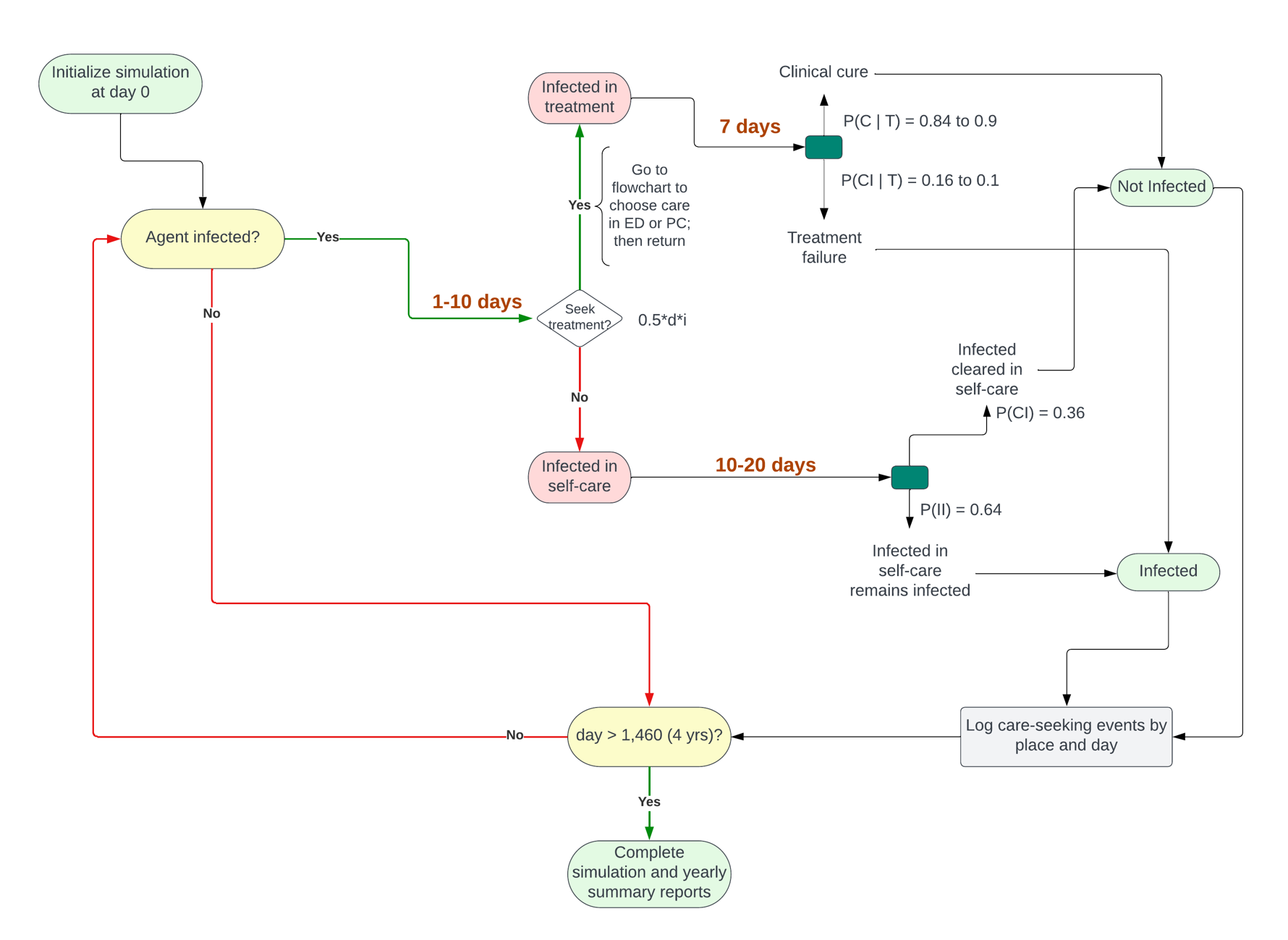
**Disease Progress and Transition Submodel**

- 1. **Health decision**: The health decision submodel defines how individuals decide whether to seek treatment for their infection or self-care at home. This submodel, inspired by the HBM, includes three of the four original constructs: perceived barriers/facilitators, perceived benefits of the behavior, and perceived severity. Perceived barriers/facilitators are represented by an individual's income, insurance status/type, and care availability (a function of an ED or enough PCs being available in their MSSA and their income/resources). Perceived benefit is determined by an individual's previous infection experience (if applicable). Perceived severity is represented by how long an individual has been infected and is included in the submodel as a time decay function. It is determined by infection duration (assuming the longer an infection goes without clearing, the more complicated and severe it becomes, and the less likely someone is to continue self-care at home).

The decision process of seeking treatment or self-care at home is controlled by the parameter ***sc*** (probability of self-care). This parameter is estimated per infected agent per time step of the simulation based on agent attributes shown in **Equation 1** and then multiplied by a time decay parameter as shown in **Equation 2** (key process/submodel section). The decision to self-care or not is based on the sampling of their objective measure (*x,* where x = 1 for self-care; x = 0 for seek treatment) from a Bernoulli distribution (**Equation 3**).

$sc=\frac{e^{0 + 0.54\left( Low income \right) - 0.45\left( Insured \right) + 0.74\left( Prior \& healed \right) + 0.19(Unavailability)}}{1+e^{0 + 0.54\left( Low income \right) - 0.45\left( Insured \right) + 0.74\left( Prior \& healed \right) + 0.19(Unavailability)}}$ (1)

Logged parameter values from Table 1

### *Low income*: a binary indicator of whether an agent lives below FPL (0 = no; 1 = yes)

### *Insured*: a binary indicator of whether an agent has insurance (0 = uninsured; 1 = insured)

### *Prior & healed*: a binary indicator of whether an agent had a previous infection that healed within 14 days, regardless of whether they sought treatment or self-cared (0 = no; 1 = yes)

### *Unavailability*: a binary indicator of whether an agent lives in an MSSA without an ED or enough primary care providers (i.e., is a designated healthcare shortage area) (0 = no; 1 = yes)

### The resulting value was then multiplied by a time decay function^13^ representing perceived severity:

$d=e^{-\beta t}$ (2)

- Where $t$ is the time in days the agent has been infected, and $\beta$is the time decay parameter (0.025 ~ 2.5% reduction per day).

### The $sc*d$ value was then sampled from a Bernoulli distribution:

$x= \sim Bernoulli\left( p \right)$ (3)

- Where $p=\left( sc \right)*(d)$ and x = 1 for self-care; x = 0 for seek treatment
  1. **Seek treatment:** Once an individual decides to seek treatment for their infection, the seek treatment submodel is used to decide whether they visit a PC or ED. Decision-making processes, and branching, is informed by literature review according to the following flow chart:


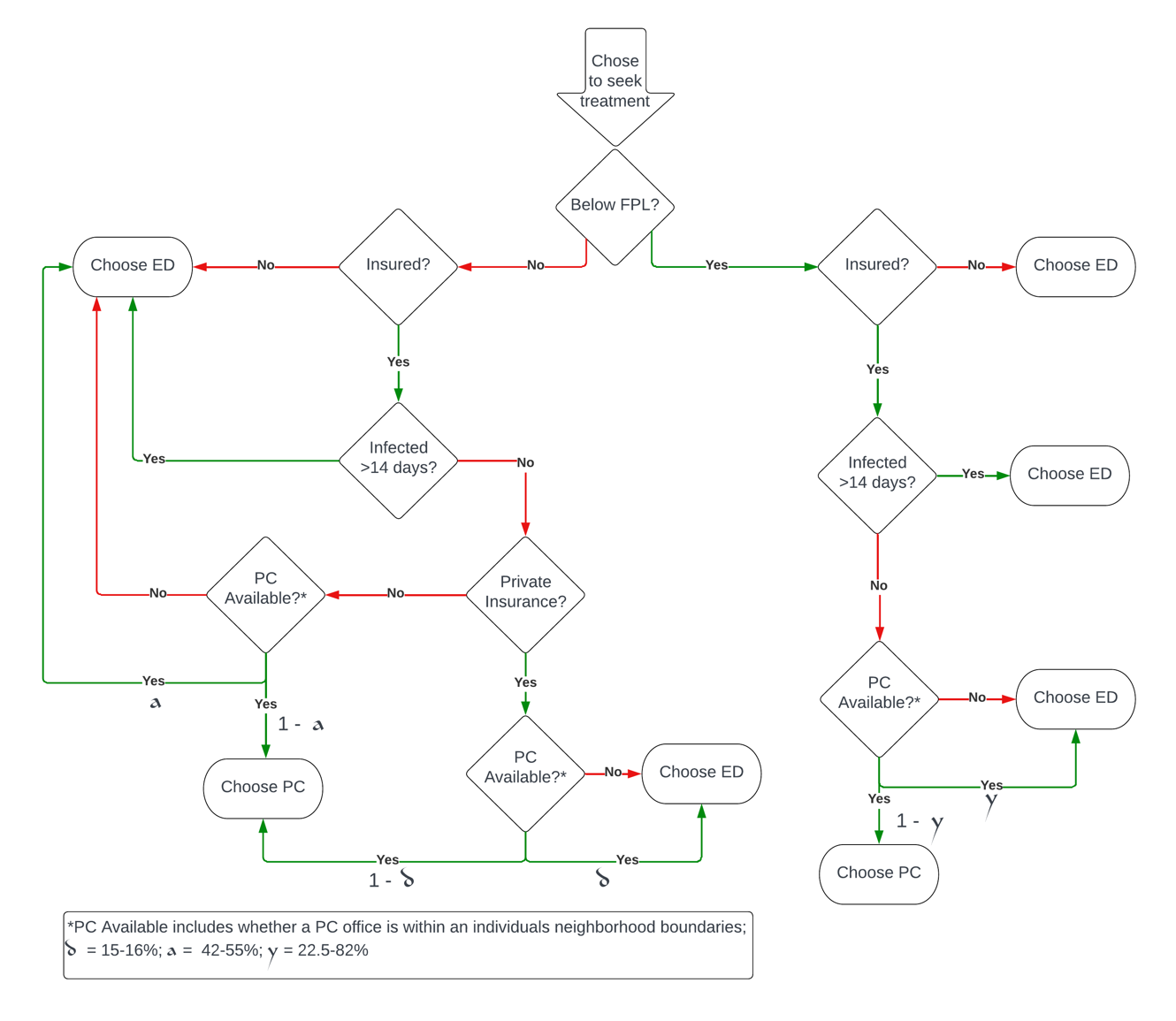
**Figure S2: Seek Treatment Submodel**

**Figures S3a-3d: Agent-based model output for CA-MRSA infection outcomes
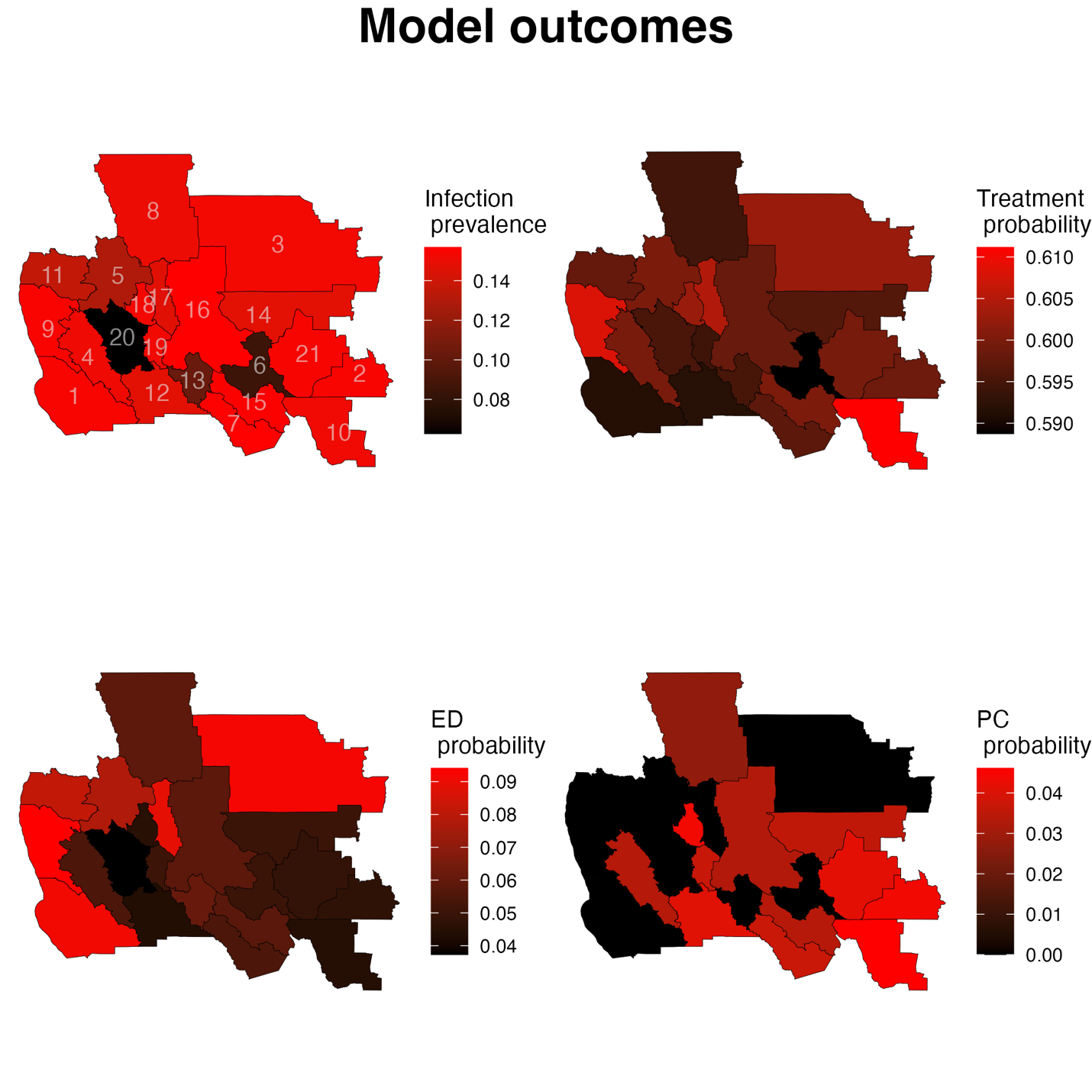
**

d)

c)

b)

a)

**Figure S4: MSSAs with statistically significantly fewer CA-MRSA cases produced from the ABM than observed in the empirical ED data as identified using Markov Chain Monte Carlo pseudo-*P-*values.**
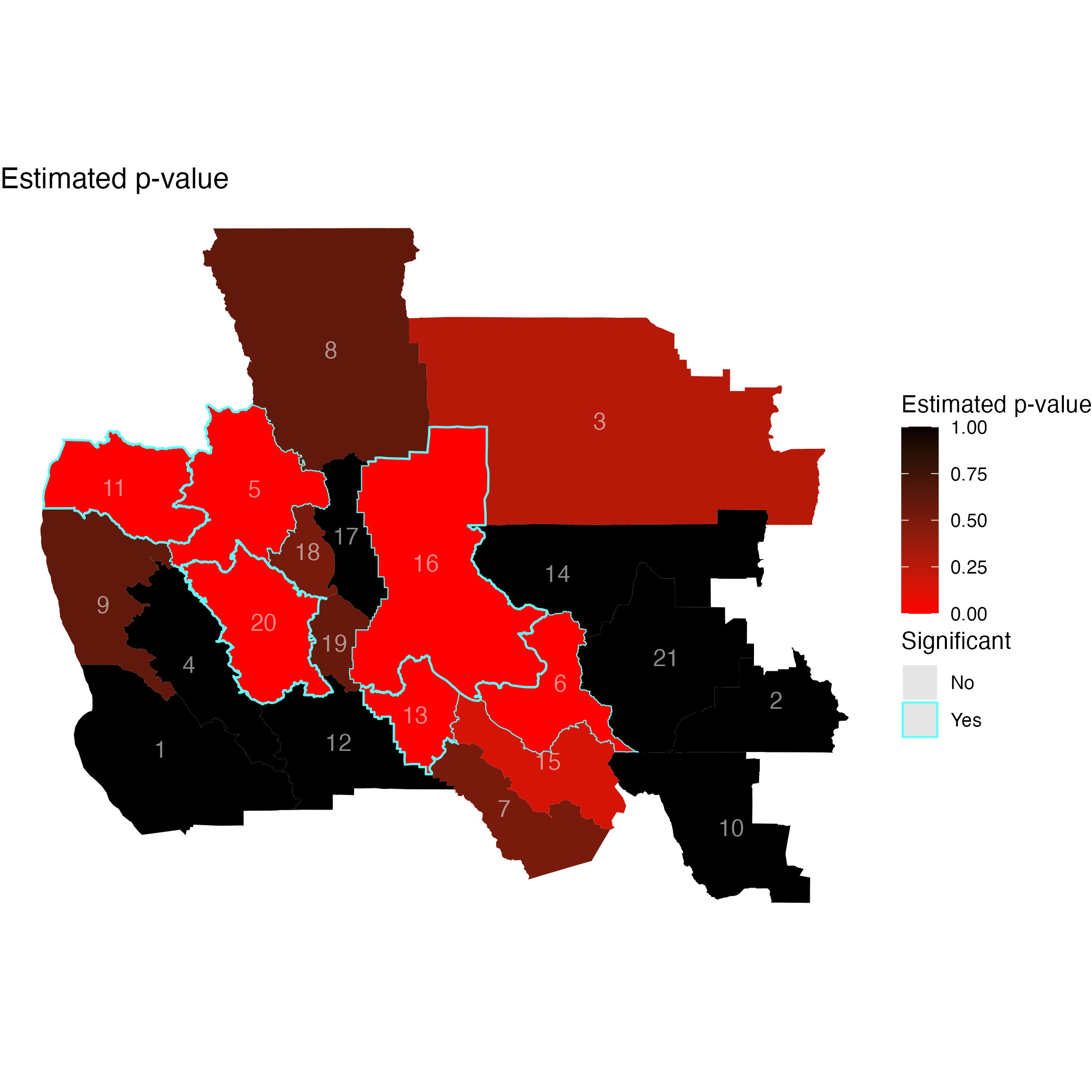


**Figures S5a-b: Results from PLS relative importance analysis**


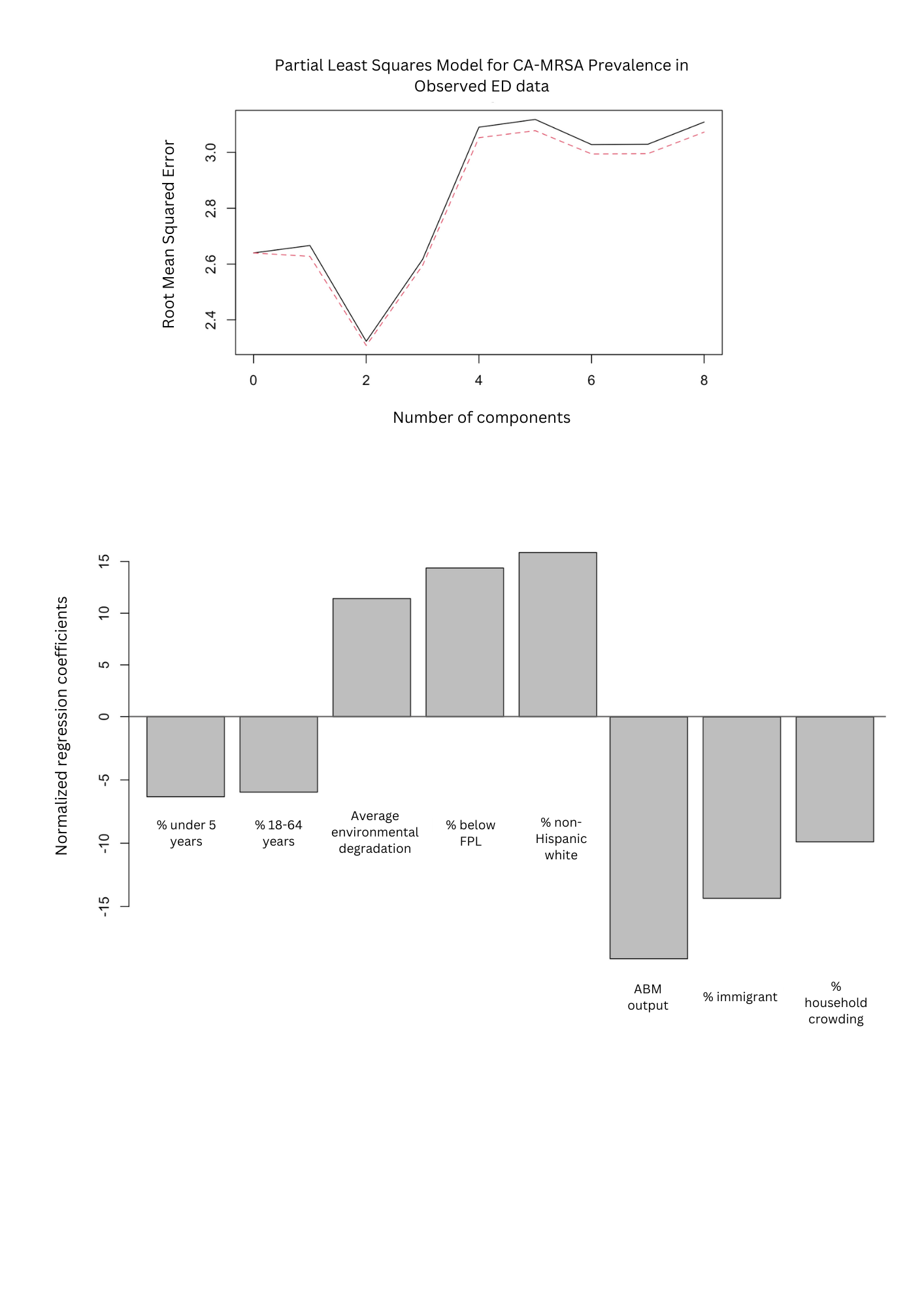


b.

a.

a) plot to identify the number of optimal components to include in the PLS model. Looking for the point in the graph where the RMSE is the lowest (2 components); b) bar chart to identify variables of relative importance in explaining variance in the outcome variable (CA-MRSA prevalence in observed ED data). The regression coefficients from the PLS are normalized, so their absolute sum is 100. *FPL = Federal poverty level; all variables are at the MSSA level.

**Figure S6: Global sensitivity analysis (GSA) for CA-MRSA infection prevalence in emergency department**


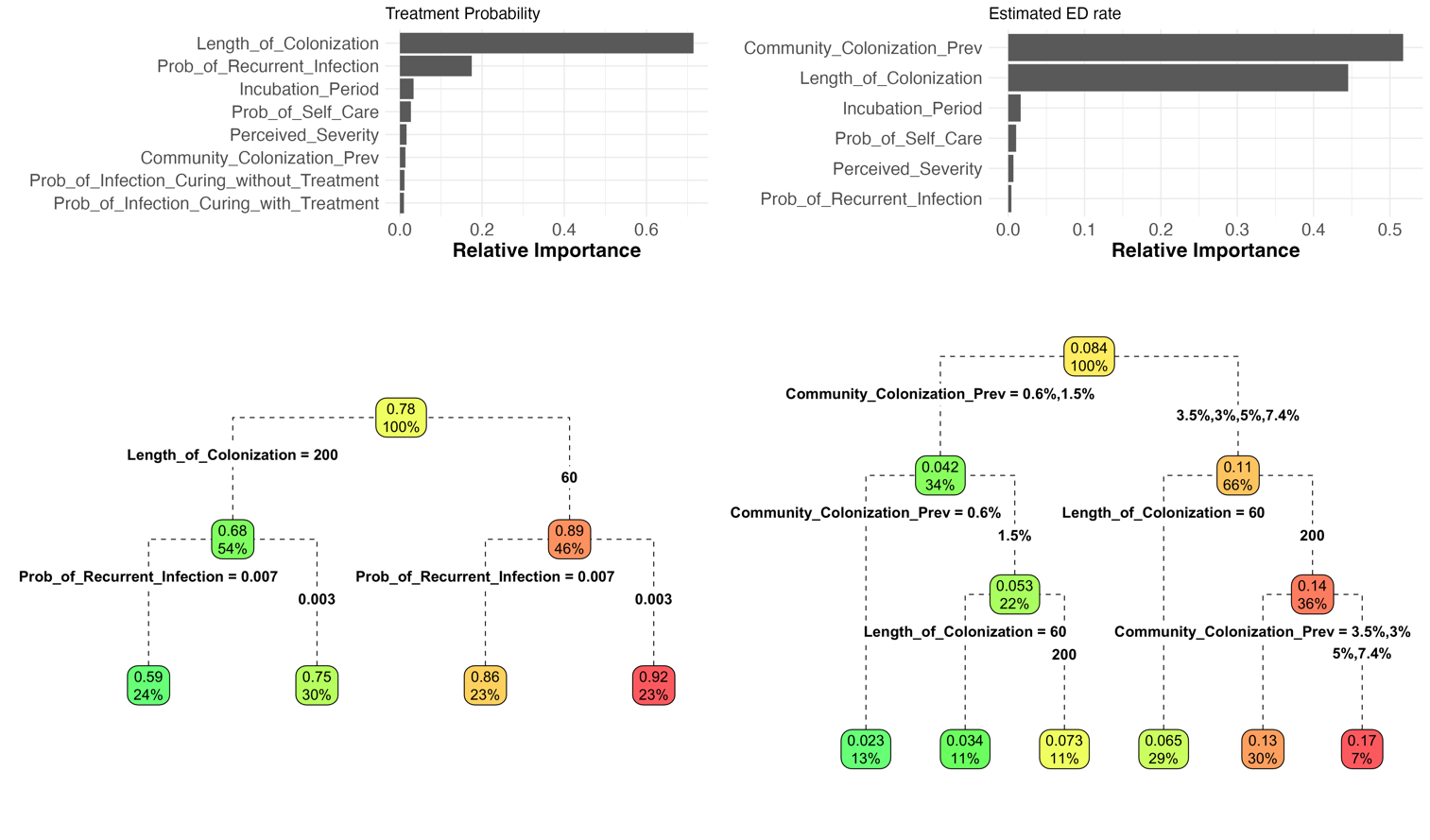


**Table S2: Characteristics of MSSAs and MSSA residents in the study area between 2016-2019**

| **MSSA Name** | **Total Population** | **Number of EDs** | **Number of Primary Care Physicians** | **HCSA** | **Number of CA-MRSA Cases** | | **Below FPL & Uninsured** | | **Above FPL & Uninsured** | | **Below FPL & Public Insurance** | | **Above FPL & Public Insurance** | | **Above FPL & Private Insurance** | |
| --- | --- | --- | --- | --- | --- | --- | --- | --- | --- | --- | --- | --- | --- | --- | --- | --- |
|  |  |  |  |  | **No.** | **Prevalence** | **No.** | **%** | **No.** | **%** | **No.** | **%** | **No.** | **%** | **No.** | **%** |
| Anchor Bay/Gualala/  Manchester/Point Arena | 4188 | 0 | 3 | No | 67 | 1.6% | 38 | 0.9% | 108 | 2.6% | 315 | 7.5% | 2521 | 60.2% | 1207 | 28.8% |
| Arbuckle/Grimes | 5647 | 0 | 0 | Yes | 79 | 1.4% | 48 | 0.9% | 363 | 6.4% | 419 | 7.4% | 2054 | 36.4% | 2762 | 48.9% |
| Artois/Elk Creek/Glenn/Grindstone  Indian Rancheria/Willows | 10668 | 1 | 9 | No | 441 | 4.1% | 111 | 1.0% | 361 | 3.4% | 1093 | 10.2% | 4165 | 39.0% | 4937 | 46.3% |
| Boonville/Navarro/Philo/Yorkville | 3023 | 0 | 1 | Yes | 85 | 2.8% | 61 | 2.0% | 137 | 4.5% | 463 | 15.3% | 1167 | 38.6% | 1195 | 39.5% |
| Brooktrails/Pine Mountain/Willits | 12690 | 1 | 11 | No | 866 | 6.8% | 187 | 1.5% | 249 | 2.0% | 1572 | 12.4% | 6387 | 50.3% | 4295 | 33.8% |
| Clearlake/Clearlake Oaks | 19553 | 1 | 15 | No | 2244 | 11.5% | 411 | 2.1% | 347 | 1.8% | 3286 | 16.8% | 10098 | 51.6% | 5412 | 27.7% |
| Cobb/Hidden Valley/Middletown | 9957 | 0 | 1 | Yes | 350 | 3.5% | 85 | 0.9% | 186 | 1.9% | 665 | 6.7% | 4590 | 46.1% | 4432 | 44.5% |
| Covelo/Dos Rios | 2674 | 0 | 3 | Yes | 127 | 4.7% | 54 | 2.0% | 171 | 6.4% | 426 | 15.9% | 1257 | 47.0% | 767 | 28.7% |
| Elk/Little River/Mendocino | 8345 | 0 | 2 | No | 250 | 3.0% | 63 | 0.8% | 189 | 2.3% | 730 | 8.7% | 3615 | 43.3% | 3749 | 44.9% |
| Esparto/Rumsey | 5722 | 0 | 1 | Yes | 103 | 1.8% | 45 | 0.8% | 174 | 3.0% | 465 | 8.1% | 1862 | 32.5% | 3176 | 55.5% |
| Fort Bragg/Westport | 12078 | 1 | 17 | No | 636 | 5.3% | 139 | 1.2% | 452 | 3.7% | 1225 | 10.1% | 6607 | 54.7% | 3656 | 30.3% |
| Hopland | 2051 | 0 | 0 | Yes | 100 | 4.9% | 31 | 1.5% | 137 | 6.7% | 278 | 13.6% | 598 | 29.2% | 1007 | 49.1% |
| Kelseyville/Lakeport | 16029 | 1 | 23 | No | 942 | 5.9% | 166 | 1.0% | 469 | 2.9% | 1560 | 9.7% | 7577 | 47.3% | 6257 | 39.0% |
| Loduga/Maxwell/Princeton/ Stonyford | 2252 | 0 | 0 | Yes | 81 | 3.6% | 32 | 1.4% | 71 | 3.2% | 261 | 11.6% | 888 | 39.4% | 1000 | 44.4% |
| Lower Lake | 9787 | 0 | 2 | Yes | 478 | 4.9% | 114 | 1.2% | 207 | 2.1% | 1046 | 10.7% | 4531 | 46.3% | 3888 | 39.7% |
| Lucerne/Nice/Upper Lake | 8869 | 0 | 2 | Yes | 852 | 9.6% | 125 | 1.4% | 434 | 4.9% | 1193 | 13.5% | 3956 | 44.6% | 3161 | 35.6% |
| Potter Valley | 1869 | 0 | 2 | No | 53 | 2.8% | 15 | 0.8% | 89 | 4.8% | 136 | 7.3% | 689 | 36.9% | 940 | 50.3% |
| Redwood Valley | 5322 | 0 | 3 | Yes | 300 | 5.6% | 47 | 0.9% | 182 | 3.4% | 393 | 7.4% | 1858 | 34.9% | 2842 | 53.4% |
| Talmage | 4097 | 0 | 1 | Yes | 218 | 5.3% | 41 | 1.0% | 92 | 2.2% | 412 | 10.1% | 1684 | 41.1% | 1868 | 45.6% |
| Ukiah | 27345 | 1 | 35 | No | 1918 | 7.0% | 366 | 1.3% | 1116 | 4.1% | 2855 | 10.4% | 11114 | 40.6% | 11894 | 43.5% |
| Williams | 5963 | 0 | 1 | Yes | 111 | 1.9% | 41 | 0.7% | 385 | 6.5% | 326 | 5.5% | 2433 | 40.8% | 2777 | 46.6% |

^ED = Emergency Department; HCSA = Healthcare Shortage Area; CA-MRSA = Community-acquired Methicillin-resistant^ *^Staphylococcus aureus^*^; FPL = Federal poverty level; MSSA characteristics from HCAi data and population totals/percentages from the US Censu^

1. Becker MH. The Health Belief Model and Sick Role Behavior. *Health Education Monographs*. 1974;2(4):409-419. doi:10.1177/109019817400200407

2. Wilder JR, Wegener DT, David MZ, Macal C, Daum R, Lauderdale DS. A national survey of skin infections, care behaviors and MRSA knowledge in the United States. *PLoS One*. 2014;9(8):e104277. doi:10.1371/journal.pone.0104277

3. Turner NA, Sharma-Kuinkel BK, Maskarinec SA, et al. Methicillin-resistant Staphylococcus aureus: an overview of basic and clinical research. *Nat Rev Microbiol*. 2019;17(4):203-218. doi:10.1038/s41579-018-0147-4

4. Macal CM, North MJ, Collier N, et al. Modeling the transmission of community-associated methicillin-resistant Staphylococcus aureus: a dynamic agent-based simulation. *Journal of Translational Medicine*. 2014;12(1):124. doi:10.1186/1479-5876-12-124

5. Weintrob A, Bebu I, Agan B, et al. Randomized, Double-Blind, Placebo-Controlled Study on Decolonization Procedures for Methicillin-Resistant Staphylococcus aureus (MRSA) among HIV-Infected Adults. *PLOS ONE*. 2015;10(5):e0128071. doi:10.1371/journal.pone.0128071

6. Agha M. Epidemiology and Pathogenesis of C. difficile and MRSA in the Light of Current NHS Control Policies: A Policy review. *Annals of Medicine and Surgery*. 2012;1:39-43. doi:10.1016/S2049-0801(12)70012-2

7. David MZ, Daum RS. Community-associated methicillin-resistant Staphylococcus aureus: epidemiology and clinical consequences of an emerging epidemic. *Clin Microbiol Rev*. 2010;23(3):616-687. doi:10.1128/CMR.00081-09

8. Fritz SA, Camins BC, Eisenstein KA, et al. Effectiveness of Measures to Eradicate Staphylococcus aureus Carriage in Patients with Community-Associated Skin and Soft Tissue Infections: A Randomized Trial. *Infect Control Hosp Epidemiol*. 2011;32(9):872-880. doi:10.1086/661285

9. Larsson AK, Gustafsson E, Nilsson AC, Odenholt I, Ringberg H, Melander E. Duration of methicillin-resistant Staphylococcus aureus colonization after diagnosis: a four-year experience from southern Sweden. *Scand J Infect Dis*. 2011;43(6-7):456-462. doi:10.3109/00365548.2011.562530

10. Liu J (Jason). Health Professional Shortage and Health Status and Health Care Access. *Journal of Health Care for the Poor and Underserved*. 2007;18(3):590-598. doi:10.1353/hpu.2007.0062

11. McConville S, Danielson C, Hsia R. *Emergency Department Use in California: Demographics, Trends, and the Impact of the ACA*. Public Policy Institute of California; 2019. Accessed October 4, 2023. https://www.ppic.org/publication/emergency-department-use-in-california-demographics-trends-and-the-impact-of-the-aca/

12. Powers D, Robinson S, Berchick E, et al. *Evaluating the Utility of Emergency Department Encounter Data and Examining Social Determinants of Emergency Department Utilization in Utah*. U.S. Census Bureau; 2021.

13. Yang Y, Diez Roux AV, Auchincloss AH, Rodriguez DA, Brown DG. A spatial agent-based model for the simulation of adults’ daily walking within a city. *Am J Prev Med*. 2011;40(3):353-361. doi:10.1016/j.amepre.2010.11.017
